# Supplementary material for: Evaluation of Streptococcus pneumoniae as a cause of acute otitis media in Colombia: A prospective study
Source: PLoS One. 2025 Jul 28;20(7):e0326660. doi: 10.1371/journal.pone.0326660 (PMC12303261; doi:10.1371/journal.pone.0326660)
Supplement: S1 Text — (DOCX) [file pone.0326660.s001.docx]

Informed consent for the project “Determination of Streptococcus pneumoniae as the main cause of acute otitis media in young children in Colombia: A prospective study”.

Hospital Infantil Napoleón Franco Pareja

In this document I express my consent for the participation of my son/daughter/represented in the project “Determination *of Streptococcus pneumonioe as the main cause of acute otitis media in young children in Colombia,* a prospective study”, sponsored by MSD Colombia, a subsidiary of Merck & Co., Inc. I am aware that the project will last 16 months, when participants will be recruited for 12 months.

The objectives of this research have been explained to me, which include:

- To describe the clinical and demographic characteristics of bacterial Acute Otitis Media (AOM) in children aged 3-59 months in Colombia.
- To determine the prevalence of bacterial AOM caused by *S. pneumoniae* and the distribution of serotypes.
- To identify antimicrobial resistance patterns of *S. pneumoniae* from middle ear fluid samples (MEF) collected from children diagnosed with AOM.

I am knowledgeable about the procedures and activities needed to perform it, the benefits and possible risks. I know that your results will help my knowledge about the clinical and demographic characteristics of bacterial AOM in children aged 3-59 months. I have also been informed that the tests and procedures will be performed by expert personnel, will be at no cost to me, and their results will be confidential. I will not receive financial compensation for my child's participation in this study.

I understand that this investigation is of minimal risk, and that the procedures to be performed are part of my child's health care. Middle ear fluid/liquid sampling or tympanocentesis is performed if my child's medical condition requires it and will be part of the diagnosis and management of my son/daughter/represented's condition.

I have been assured that any questions or concerns I have about the procedures, risks, benefits and other matters related to the research can be directed to the principal investigator Wilfrido Coronell Rodriguez at XXXXX or the email wiIfridocoroneIl@gmaiI.com or to the co-investigator Alejandra Puerto at phone number XXXXX or the email apuerto@alzak.com.co.

The investigators have explained to me that I can withdraw my consent at any time and stop participating in the study without prejudice to my legal rights, reputation, or access to health care. If I withdraw from the study, the samples obtained will not be further analyzed and will not be used to develop new tests (future studies). If samples continue to be stored, they can only be analyzed at the request of the regulatory entity.

If you have any questions about your rights as a research participant, you may contact a representative of the Ethics Committee at 7848997 Ext. 4001 or send a letter to: IMAT oncomédica's

Ethics Committee to the following e-mail address comitedeeticaeinvestigaciones@gmail.com

On a voluntary basis and without any coercion, I declare my willingness to contribute to the necessary activities for the realization of this research and therefore I authorize:

- Answer a questionnaire that will contain social and clinical information about the patient at the time of medical care.
- The use of the middle ear discharge sample obtained during health care for the identification of the etiologic agent of ear infection, as well as the serotyping of the agent *S. pneumoniae*.
- That I be contacted by telephone within 10 days of the sample collection to evaluate the clinical evolution of my daughter/son/represented.
- Answer a questionnaire that will contain clinical information about the evolution of the patient's clinical condition.

At the time of health care, the treating physician will apply a questionnaire containing the patient's social and clinical information. This information will be stored in a database, to which only the study investigators will have access.

The sample obtained will be transported to the clinical laboratory of the Napoleón Franco Pareja Children's Hospital, where the possible germ causing the infection will be identified. If *S. pneumoniae* is identified, the germ sample will be sent to the National Institute of Health to identify the serotype.

Ten days after medical care, the participant will be contacted, and a second questionnaire will be carried out by telephone to know the clinical evolution. This information will be stored for study analyses.

This project was reviewed and approved by the Ethics Committee of IMAT Oncomédica and all its protocols are in accordance with international laws and the provisions in force in Colombia according to Resolution No. 008430 of 1993, which establishes the scientific, technical and administrative standards for health research.

MOTHER

NAME:

SIGNATURE:

DATE:
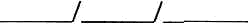


FATHER

NAME:

SIGNATURE:

DATE:
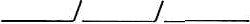


LEGAL REPRESENTATIVE (IF APLICABLE)

NOMBRE:

SIGNATURE:


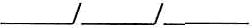
DATE:

RESEACHER

NAME:

SIGNATURE:

DATE:
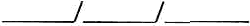


WITNESS 1

NAME:

SIGNATURE:

DATE:
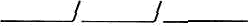
 KINSHIP:

ADDRESS:

WITNESS 2

NAME:

SIGNATURE:

DATE:
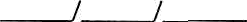
 KINSHIP:

ADDRESS:
