# Supplementary material for: Evaluation of Streptococcus pneumoniae as a cause of acute otitis media in Colombia: A prospective study
Source: PLoS One. 2025 Jul 28;20(7):e0326660. doi: 10.1371/journal.pone.0326660 (PMC12303261; doi:10.1371/journal.pone.0326660)
Supplement: Attachment — Submitted filename: Response to Reviewers.pdf [file pone.0326660.s003.pdf]

Luis Felipe Reyes, MD, MSc. PhD
Academic editor
PLOS ONE

Dear Dr. Reyes and reviewers,

Thank you for your comments and valuable suggestions. We are sure that those have allowed us to improve our manuscript considerably. The modifications introduced in the manuscript were done as required. All recommendations were implemented and are highlighted in red throughout the text. Detailed responses to the journal requirements and reviewer's comments are presented as follows:

Journal requirements:

1. Please ensure that your manuscript meets PLOS ONE's style requirements, including those for file naming. The PLOS ONE style templates can be found at https://journals.plos.org/plosone/s/file?id=wjVg/PLOSOne_formatting_sample_main_body.pdf and https://journals.plos.org/plosone/s/file?id=ba62/PLOSOne_formatting_sample_title_authors_affiliations.pdf

   R/ Thank you. We have reviewed PLOS ONE's style requirements and have adjusted the manuscript to conform to them.

2. Please note that funding information should not appear in any section or other areas of your manuscript. We will only publish funding information present in the Funding Statement section of the online submission form. Please remove any funding-related text from the manuscript.

   R/ Funding information was removed from the manuscript

3. Please state what role the funders took in the study. If the funders had no role, please state: "The funders had no role in study design, data collection and analysis, decision to publish, or preparation of the manuscript." If this statement is not correct you must amend it as needed. Please include this amended Role of Funder statement in your cover letter; we will change the online submission form on your behalf.

   R/ This study was funded by MSD Colombia, a subsidiary of Merck & Co., Inc., Rahway, NJ, USA. Claudia Beltran, Sebastian Medina, Monica Maria Rojas, Maria Alejandra Betancur and Luis Enrique Farias are MSD employees. This did not interfere with the authors' ability to analyze, interpret the data, or prepare the manuscript.
   We updated the cover letter considering this new statement.

4. We note that one or more of the authors are employed by a commercial company: Merck Sharp & Dohme Corp.
   A. Please provide an amended Funding Statement declaring this commercial affiliation, as well as a statement regarding the Role of Funders in your study. If the funding organization did not play a role in the study design, data collection and analysis, decision to publish, or preparation of the manuscript and only provided financial support in the form of authors' salaries and/or research materials, please

review your statements relating to the author contributions, and ensure you have specifically and accurately indicated the role(s) that these authors had in your study. You can update author roles in the Author Contributions section of the online submission form. Please also include the following statement within your amended Funding Statement. “The funder provided support in the form of salaries for authors [insert relevant initials], but did not have any additional role in the study design, data collection and analysis, decision to publish, or preparation of the manuscript. The specific roles of these authors are articulated in the ‘author contributions’ section.” If your commercial affiliation did play a role in your study, please state and explain this role within your updated Funding Statement.

B. Please also provide an updated Competing Interests Statement declaring this commercial affiliation along with any other relevant declarations relating to employment, consultancy, patents, products in development, or marketed products, etc. Within your Competing Interests Statement, please confirm that this commercial affiliation does not alter your adherence to all PLOS ONE policies on sharing data and materials by including the following statement: "This does not alter our adherence to PLOS ONE policies on sharing data and materials.” (as detailed online in our guide for authors http://journals.plos.org/plosone/s/competing-interests). If this adherence statement is not accurate and there are restrictions on sharing of data and/or materials, please state these. Please note that we cannot proceed with consideration of your article until this information has been declared. Please include both an updated Funding Statement and Competing Interests Statement in your cover letter. We will change the online submission form on your behalf.

A. Thank you for this commentary. We already provided and amended the Funding Statement, as follows: “*This study was funded by MSD Colombia, a subsidiary of Merck & Co., Inc., Rahway, NJ, USA. Claudia Beltran, Sebastian Medina, Monica Maria Rojas, Maria Alejandra Betancur and Luis Enrique Farias are MSD employees. This did not interfere with the authors' ability to analyze, interpret the data, or prepare the manuscript.*”

B. Thanks for your suggestion. We provided an updated Competing Interests Statement, as follows: “*This study was funded by MSD Colombia, a subsidiary of Merck & Co., Inc., Rahway, NJ, USA. Claudia Beltran, Sebastian Medina, Monica Maria Rojas, Maria Alejandra Betancur and Luis Enrique Farias are MSD employees. This does not alter our adherence to PLOS ONE policies on sharing data and materials”*

5. In the online submission form, you indicated that “Data cannot be shared publicly because of sensitive information. Data will be shared upon request.” All PLOS journals now require all data underlying the findings described in their manuscript to be freely available to other researchers, either 1. In a public repository, 2. Within the manuscript itself, or 3. Uploaded as supplementary information. This policy applies to all data except where public deposition would breach compliance with the protocol approved by your research ethics board. If your data cannot be made publicly available for ethical or legal **reasons (e.g., public availability would compromise patient privacy), please explain your reasons** on resubmission and your exemption request will be escalated for approval.

   R/ We appreciate your commentary in terms of the PLOS data policy. However, sharing the information in a public repository could jeopardize informed consent approved by the Ethics Committee of IMAT Oncomédica (ONC-CEI-CEI-231-2022).

Explicitly the informed consent says: "*I have also been informed that the tests and procedures will be performed by expert personnel, will be at no cost to me, and* ***their results will be confidential***", which implies that if we potentially share the database, we will be braking the agreement stablished with the patients and their families.
The above support the journal policy which stablish that "This policy applies to all data except where public deposition would breach compliance with the protocol approved by your research ethics board".

6. PLOS requires an ORCID iD for the corresponding author in Editorial Manager on papers submitted after December 6th, 2016. Please ensure that you have an ORCID iD and that it is validated in Editorial Manager. To do this, go to 'Update my Information' (in the upper left-hand corner of the main menu), and click on the Fetch/Validate link next to the ORCID field. This will take you to the ORCID site and allow you to create a new iD or authenticate a pre-existing iD in Editorial Manager.

   R/ Thank you for this suggestion. We included the ORCID ID of the corresponding author in the online form. https://orcid.org/0000-0001-9458-864X ORCID NELSON ALVIS GUZMAN

7. Please ensure that you refer to Figures 2-4 in your text as, if accepted, production will need this reference to link the reader to the figure.

   R/ Thank you, all figures are referenced in the manuscript.

8. We note you have included a table to which you do not refer in the text of your manuscript. Please ensure that you refer to Table 2 and 3 in your text; if accepted, production will need this reference to link the reader to the Table.

   R/ Thank you, all figures and tables were double checked in order to ensure correctly reference into the manuscript.

Reviewers' comments:

Reviewer #1

Manuscript format and Material

1. This is an interesting paper entitled: Evaluation of Streptococcus pneumoniae as a cause of acute otitis media in Colombia: a prospective study. I want to thank the authors for their efforts in researching pneumococci. Even when the paper is well-written and has some important information, in my scope, it looks like a mix of information in different sections. The manuscript has some major aspects that must be addressed before your work is ready for publication. Grammatically it is well-written and understandable. However, it lacks an organized structure; some sections are very long and lose a common thread. A major restructure, and a new version of the discussion is needed.

   R/ Thank you for your valuable feedback on our manuscript. We acknowledge your comments regarding the manuscript's structure and the organization of information across different sections. To address this, we carefully revised the manuscript to ensure a more coherent and logical flow. We also worked on restructuring the

discussion section to enhance clarity and improve its alignment with the study's findings.

Introduction

2. The introduction looks very long and should be reorganized, with a maximum structure of 3 or 4 paragraphs. In some parts, authors seem to be throwing out ideas for discussion about the subject matter, but they are not argued and remain as free sentences. The number of paragraphs of less than 10 lines hinders the reader from jumping to ideas. Provide more information about MEF and its used in previous studies

   R/ Thank you for your valuable feedback. In response to your comments, we have shortened and reorganized the introduction to improve clarity and focus. The revised introduction is now structured into fewer paragraphs, ensuring a smoother flow and better alignment with the manuscript's objectives. Additionally, we have clarified the role of middle ear fluid (MEF) analysis in AOM research and provided relevant information to address the reviewer's request (Pages 3-4; lines 52-103).

3. The initial sentence in paragraph 4 should be modified. Also, reference number 4 does not support the sentence; there are many different pathways related to the development of SPN AOM. SPN colonization is considered a prerequisite, but there is some controversial data in this regard.

   R/ Thank you for recommendation. We have carefully reviewed the references and incorporated new references that better support the statement regarding the pathways involved in SPN AOM development and the role of SPN colonization as a prerequisite, acknowledging the existing controversy in the literature (Pages 3-4; lines 52-103).

4. Paragraph 5 does not include meningitis as a complication; relevant data about SPN AOM igniting invasive pneumococcal disease is available. this paragraph is weak; it lacks data on vaccination, vaccination coverage, and circulating serotypes. Colombian literature on these topics has been written by authors such as Severiche et al. and Serrano et al.

   R/ After updating introduction and shorten its length, paragraph 2 mentions meningitis as a complication "*Among its complications, otomastoiditis is the most prevalent, although severe conditions such as meningitis and encephalitis have also been documented (9).* In paragraph 3, data on vaccination, vaccination coverage, and circulating serotypes is presented (Page 4; lines 70-83).

5. Paragraph 6 mentioned several studies, but the references do not match. You should revise the metanalysis in this regard. There is so much information in children rather than in adults. The section that refers to the SPN capsular switch needs more data and support.

   R/ Thank you for your insight. Since introduction has been shortened, the order of paragraphs has changed. Meningitis as a complication is now mentioned in paragraph 2. Paragraph 3 contains relevant and precise information about SPN vaccination coverage. The suggested references have been added to the paragraphs (Page 4; lines 70-83).

6. Paragraph 7, please include the rates of effective vaccination in Colombia with the previous two vaccines. To provide background information to the reader about which vaccine provided "protection" while the study was being performed.

   R/ The paragraph has been revised, and we have incorporated vaccination coverage rates to provide a clearer epidemiological context. The revised text now states:

   "In 2011, PCV10 was incorporated into the national immunization program and became universally available in 2012, reaching 89% coverage (23,24). The introduction of PCV-10 led to an 84.7% national reduction in IPD due to vaccine serotypes in children under five years of age (25,26). By 2019, national vaccination coverage reached >90%, although significant variability was observed among departments; for instance, in Bolívar, S. pneumoniae vaccination historically lagged behind national rates, reaching 80–89% coverage in 2021." (Page 4; lines 77-83).

7. Paragraph 8: Please provide the numbers for the decrease in incidence.

   R/ The paragraph has been revised to ensure accuracy regarding decrease in incidence of IPD in Colombia as follows:

   "The introduction of PCV-10 led to an 84.7% national reduction in invasive pneumococcal disease (IPD) due to vaccine serotypes in children under five years of age" (Page 4; lines 78-80).

   Methods

8. Please provide clear information about the type of study. Is this a monocentric study? (provide a line saying so)

   R/ We appreciated your suggestion. The sentence has been modified, as follows:

   "This prospective observational cohort single-center study included children aged 3 to 59 months diagnosed with AOM who consulted the Napoleon Franco Pareja Children's Hospital in Cartagena, Colombia" (Page 5; lines 105-106).

9. What kind of guidelines were followed to perform a study on children? Please provide some lines in the first paragraph of methods standing the Helsinki declaration, the informed consent process, and the approval of an ethical committee. Consider moving the ethical considerations section after the first paragraph as this is a study performed in children.

   R/ Thanks, we have moved the ethical considerations section after the first paragraph of methods. Also, we have added this sentence

   "*All study procedures were implemented in compliance with the Helsinki Declaration*" and additional supplementary information about consent informed form (Page 6; lines 110-121).

10. Tympanocentesis is an invasive procedure with several risks, I would like the authors to provide supplemental information with a copy of the informed consent.

R/ We understand your inquiry. However, Tympanocentesis was not routinely done in all cases, it was only performed in one patient who presented clinical complications and who also had positive Streptococcus pneumoniae. Also, we added informed consent form as supporting information (See S1 Text. Informed consent form) (Page 6; line 116; Page 15 lines 270-272).

11. How did the authors avoid reporting a false SPN such as Streptococcus pseudonemoniae, which usually shows alpha hemolysis halo and sensitivity to optokine?

R/ Thank you very much for this excellent question. Although *Streptococcus pseudopneumoniae*, is a different species from *Streptococcus pneumoniae*, shows a similarity of almost 50% with *pneumococcus*. To avoid reporting false *pneumococcus*, the following was done:

Phenotypic tests were performed such as:
1. The optochin test: the strains that showed alpha-hemolysis were inoculated on sheep blood agar in a CO2 incubator (5-10%) and a 5 mg optochin disk was placed and incubated for 18-24 hours at a temperature of 37ºC. After this, the strains were reviewed and those that had an inhibition halo > 14 mm (sensitive to optochin) confirmed the presence of *pneumococcus*, since *streptococcus pseudopneumoniae* is resistant to optochin when its strains are exposed to a CO2 atmosphere of 5-10%. For this reason, we did not leave the strains incubating with optochin in a normal atmosphere because then *streptococcus pseudopneumoniae* is sensitive to optochin.

2. The other phenotypic test used was bile solubility: The isolated strains were exposed to bile salts and these were lysed or destroyed, i.e. giving a positive bile solubility test.
Streptococcus pseudopneumoniae are insoluble in bile.
After this, the strains that tested positive for these two phenotypic tests were processed by automated methods for confirmation and susceptibility profile and were confirmed as *Streptococcus pneumoniae*. In addition, the strains were sent to the National Institute of Health for serotyping by Quellum test and were also confirmed as Streptococcus pneumoniae. This information was added in the method section (see Page 8-9; lines 167-173).

12. How was the value of patients to be included estimated?

R/ As this was a prospective study, we aimed to include all eligible patients within the predefined timeframe, ensuring a representative sample of the target population. The selection was guided by previous epidemiological data on AOM incidence and the expected prevalence of *Streptococcus pneumoniae* as an etiological agent in similar settings.

Figures & Tables

13. It is important to include in figure 1 the number of patients screened.

R/ We appreciate the reviewer's suggestion to include the number of patients screened in Figure 1. However, hospital patient data are restricted due to confidentiality policies. In accordance with these regulations, only patients who met the inclusion criteria were reported by the emergency medical doctors and included in the study.

As a result, we are unable to provide the total number of patients with AOM who did not meet the inclusion criteria.

Results

14. There are several typos repeatedly in the results “(Error! Reference source not found.)” and There is a typo in the first paragraph of results (after ... April 2023…)

    R/ Thanks, they were adjusted in the manuscript.

15. Only 6 SPN cases among 61 patients were recruited. What was the etiology for the other 55?

    R/ In the manuscript, we have already detailed the etiology of all 61 AOM cases, including both *Streptococcus pneumoniae*-positive and other findings. Among the 61 middle ear fluid samples, *S. pneumoniae* was isolated in 6 cases (9.8%), while *Pseudomonas aeruginosa* (14.8%) and methicillin-resistant *Staphylococcus aureus* (13.1%) were the most frequently identified pathogens. Additionally, a considerable proportion of samples yielded only ear commensal pathogens, which is consistent with previous studies where AOM cases often present without bacterial pathogens due to viral etiologies or prior antibiotic use. These findings are detailed in the Results section (Table 1, Figure 3) (Page 8; lines 259-267).

16. I suggest that the authors review the reporting structure of the results, use simple numerators and denominators when referring to percentages to make it easier to read, e.g. : XX/XX (XX%)

    R/ Thank you for your suggestion. We have reviewed the reporting structure of the results and used simple numerators and denominators when referring to percentages to improve readability.

Discussion

17. The discussion must be reorganized and reformulated. The aspects to be discussed should be contrasted with external or internal data previously found before the PCV-10 vaccine introduction.

    R/ We appreciate the reviewer’s insightful suggestion regarding the discussion section. We have now reorganized and reformulated the discussion to improve clarity and coherence. Specifically, we have added a more structured contrast between our findings and both external and internal data collected before the introduction of the PCV-10 vaccine. This includes comparisons with national and international studies on the prevalence of AOM and serotype distribution prior to PCV-10 implementation. These adjustments provide a clearer understanding of the vaccine’s impact on the epidemiology of AOM in Colombia (see lines 300-309; 319-325).

18. It is ambitious to say that this single-center study of 62 patients describes the effect of vaccination in children suffering from otitis when 1 in 4 children who consult for this possible diagnosis. Likewise, it is estimated that 40% of the consultations are for AOM in children, and this cut-off seems small and even more so when it is mentioned that the hospital where it was carried out is the one that provides the most care to patients from both the private and public sectors.

R/ We appreciate the reviewer's concern regarding the scope of our study and the generalizability of our findings. We acknowledge that our study is a single-center analysis with a limited sample size; however, our objective was not to provide a comprehensive assessment of the overall impact of vaccination on AOM incidence but rather to describe the clinical and microbiological characteristics of AOM cases caused by *S. pneumoniae* in a vaccinated pediatric population.

Additionally, while we recognize that AOM represents a significant proportion of pediatric consultations, our study focused on cases with microbiological confirmation, which inherently limits the sample size. The recruitment process followed strict inclusion criteria, and only patients confirmed with AOM by an otorhinolaryngologist were included, ensuring diagnostic accuracy.

To address this concern, we have refined our discussion to better contextualize the study's findings within its scope and limitations. We now explicitly state that our results provide a baseline for future larger-scale, multi-center studies and contribute valuable microbiological surveillance data on *S. pneumoniae* serotypes post-PCV-10 introduction (Page 23; lines 423-425).

19. The strengths of the study should be focused on supporting the results by using high-quality techniques, standard procedures, and double-checked test were performed, not in the team training.

    R/ Thank you for your insightful suggestion. We have revised the Strengths section to focus on the high-quality laboratory techniques, standardized procedures, and double-checking of tests rather than emphasizing team training. Specifically, we now highlight the use of validated microbiological methods, strict adherence to sample handling protocols, and cross-verification of results between the hospital laboratory and the Colombian National Institute of Health (INS, national laboratory of reference) to ensure data accuracy and reliability (Page 22; lines 401-408).

Reviewer #2

Introduction

1. In the introduction, the sentence: "In Latin America, the incidence of AOM ranges from 4.25% to 6.78% [2]. In a 2016 study published in a Caribbean city in Colombia, the incidence of AOM was found to be 29.4% per 1,000 children [3]. This poses a challenge for public health and health economics" It is recommended that the values be presented in the same units to facilitate comparison, either as percentages or as rates per 1,000 children. Review the text "29.4% per 1,000 children," as the "%" sign is unnecessary.

   R/ Thank you for your recommendations. The % was eliminated after 29.4 value as you suggested (Page 3, line 57).

2. In the introduction, review the sentence: "during the early introduction of PCV-7/PCV-13 (2003-2005), and in the late period after the introduction of PCV-7/PCV-13 (2006-2009)". PCV13 was not available during those time periods.

   R/ We appreciate the clarification regarding the availability of PCV-13 during the specified time periods. The paragraph was shortened, and it is written now as follows:

"After the introduction of PCV-7 and PCV-13 in the United States, studies reported significant declines in AOM caused by serotypes included in the vaccines, although non-vaccine serotypes like 19A exhibited replacement effects" (Page 4, lines 71-74).

3. In the introduction, review the sentence: "In Colombia, the PCV-7 vaccine (Prevnar®) was introduced in the Expanded Program on Immunization (EPI) in Bogotá D.C. in 2009 with a '2+1' schedule at 2, 4, and 12 months of age. It was implemented throughout the country during 2010 and 2011". Other studies report different dates for the implementation of PCV7 in Colombia: "In Colombia, vaccination against pneumococcus began in 2006 with PCV7, initially targeting children under 2 years of age at high risk of IPD. Since 2008, the vaccine was administered universally initially in Bogotá, and since 2009 it was extended to the departments of Colombia with the highest mortality from acute respiratory infection. In the 2010–2011 period, given the withdrawal of PCV7 from the market, PCV13 was administered in Bogotá and in the higher risk departments". Ref: Camacho-Moreno G, Leal AL, Patiño-Niño J et al. Serotype distribution, clinical characteristics, andantimicrobial resistance of pediatric invasive pneumococcal disease in Colombia during PCV10 mass vaccination (2017–2022). Front. Med 2024, 11:1380125.doi: 10.3389/fmed.2024.1380125. The implementation dates of PCV10 and PCV13 are the same as those reported in other studies from Colombia.

   R/ We appreciate this observation, and the reference provided. We have reviewed the information and adjusted the sentence in our manuscript to align with the reported dates of PCV-7 implementation in Colombia, as documented in the cited study, as follows:

   "*In Colombia, PCV-7 was introduced in 2006, for high-risk children under 2 years of age and was expanded in Bogotá in 2008. In 2009, coverage extended to high-mortality regions, and between 2010 and 2011, PCV13 replaced PCV7 nationwide. In 2011, PCV10 was incorporated into the national immunization program and became universally available in 2012*" (Page 4; lines 74-78).

Results

4. A total of 61 patients were included in the study. How many cases of otitis media occurred in the hospital during the study period? What proportion of patients who presented with AOM were included in the study?

   R/ As previously mentioned, hospital patient data are restricted due to confidentiality policies. In accordance with these regulations, only patients who met the inclusion criteria were reported by emergency medical doctors and included in the study. Therefore, we are unable to provide the total number of AOM cases that occurred in the hospital during the study period, or the proportion of patients included.

5. delete the phrases: "Error! Reference source not found.Error! Reference source not found"
   R/ Thanks, they were deleted.

6. I recommend describing in the results the type of pneumococcal vaccine received by the 5 patients who were vaccinated. The phrase: "Of the six cases with *S. pneumoniae* isolation, five had received PCV-10 given that they were born before April 30, 2022" in the discussion should be moved to the results section. It is also recommended to

describe, in the results section, the vaccines used in patients who experienced non-pneumococcal otitis.

R/ The pneumococcal valence vaccine received by all 61 patients with reported vaccination status has been described, and this information has been moved to the Results section, in line with your recommendation, as follows:

"Page 21, Line 375: *Regarding the clinical characteristics of the patients (Table 2): of the 61 patients, 38 (62.3%) received the PCV-10 vaccine, as they were born before April 30, 2022. 20 (32.8%) received the PCV-13 vaccine, and 3 (4.9%) did not receive any pneumococcal vaccine. Among the unvaccinated patients, one tested positive for S. pneumoniae and presented with clinical complications. Among the six patients with S. pneumoniae isolation, five (83.3%) had received PCV-10, while the remaining case (16.7%) corresponded to the unvaccinated patient mentioned above.*"

7. "Regarding resistance patterns, Figure indicates that all pneumococcal serotypes isolated showed some degree of antimicrobial resistance" Add the corresponding figure number.

   R/ Thank you, it was adjusted (Page 15; line 277).

Figures & Tables

8. Figure 1 indicates that 82 patients were candidates; I recommend including this information in the main text.

   R/ Thank you for your recommendation. This information was included in the first paragraph of the results section (Page 11; lines 222-225).

9. How many patients had negative cultures? In table 2 "55 AOM patients, microorganism other than *S. pneumoniae*," suggesting that all cultures were positive and in Figure 3, the culture results are described, and it indicates that all cases had microbiological isolation, 18 had microbiome of the external auditory canal. However, the discussion states, "In 37% of the cultures, there was no bacterial growth, a finding similar to our study." Review this phrase.

   R/ The wording in the Discussion section of the manuscript has been revised to clarify that our study did not have cultures with "no bacterial growth" but rather isolated commensal microorganisms from the external auditory canal. In contrast, the cited study summarized these findings as "no bacterial growth." This adjustment ensures consistency between our results and the interpretation presented in the discussion. Additionally, Figure 3 and Table 2 correctly reflect the microbiological findings, specifying the cases where only ear commensals were isolated (Page 19; lines 346-348).

10. Table 3. Clarify whether the beta-lactam resistance is to penicillin and/or ceftriaxone. If resistant to penicillin or ceftriaxone, it is advisable to report the MIC.

    R/ Thanks for your commentary. Table 3 has been reorganized to include MIC values and specify the antibiotics tested. The table now clearly presents the antimicrobial susceptibility data, ensuring transparency in reporting.

Discussion

11. In the discussion section the phrase: “who received PCV-10 previously. In addition” Add a period after previously.

    R/ Thank you, it was added (Page 18; lines 314-315).

12. “The findings of AOM-causing pneumococcal serotypes not included in PCV-10 (19, 10F and 35)” The serotype is 19ª

    R/ Thank you, it was adjusted (Page 18; lines 319-320).

13. Our study found that the samples exhibited a resistance pattern consistent with what has been described in the literature. This included resistance to macrolides, clindamycin, TMP/SMX, and aminoglycosides” Pneumococcus is intrinsically resistant to aminoglycosides.

    R/ This point has been corrected in the manuscript. The reference to aminoglycoside resistance has been removed, as pneumococcus is intrinsically resistant to this class of antibiotics. The clarification enhances the accuracy of our findings in alignment with existing literature.

Sincerely,

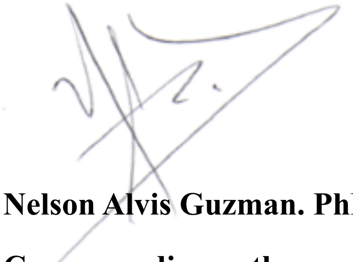

Nelson Alvis Guzman. PhD

Corresponding author
Universidad de la Costa
nalvis@cuc.edu.co
