# Supplementary material for: Evaluation of Streptococcus pneumoniae as a cause of acute otitis media in Colombia: A prospective study
Source: PLoS One. 2025 Jul 28;20(7):e0326660. doi: 10.1371/journal.pone.0326660 (PMC12303261; doi:10.1371/journal.pone.0326660)
Supplement: Attachment — Submitted filename: Response_to_Reviewers.pdf [file pone.0326660.s004.pdf]

Luis Felipe Reyes, MD, MSc. PhD
Academic editor
PLOS ONE

Dear Dr. Reyes and reviewers,

We thank the reviewers for their thorough evaluation and constructive comments. In response, we have carefully revised the manuscript to address all points raised. Below, we detail each reviewer's comment followed by our response and a summary of the changes made in the revised version of the manuscript.

Comment 1

Reviewer comment:
In line 52, a reference is missing to support this sentence.

Author response:

A reference has been added to the aforementioned line: "Gavrilovici C, Spoială EL, Miron IC, Stârcea IM, Haliţchi COI, Zetu IN, et al. Acute Otitis Media in Children—Challenges of Antibiotic Resistance in the Post-Vaccination Era. Microorganisms. 2022;10(8)."

Comment 2

Reviewer comment:
The study by Sierra Lopez and Zapata should also be included in the introduction to give specific context to the entity in question.

Author response:

We included the study by Sierra et al. in the introduction to provide specific local context regarding the etiology of otitis media in Colombia. The following sentence was added (Lines 64–66): "In Colombia, similar trends have been observed; a study conducted in Cali reported that the most commonly isolated bacteria in pediatric otitis media cases were H. influenzae (31%) and S. pneumoniae (30%) (14)."

Comment 3

Reviewer comment:

It is important to review the lines referring to the introduction of the vaccines; the flow of information varies from global to local contexts repeatedly and can confuse the reader. I suggest introducing the complete global context and ending with the local context. lines 76-77

Author response:

We have reorganized lines 73–87 to improve the clarity and flow of information about the introduction of pneumococcal vaccines. The section now begins with the global perspective and then moves on to the national context in Colombia.

Comment 4

Reviewer comment:
Line 83 has no reference.

Author response:

Reference 29 was added: Rodríguez WC, Mora-Salamanca AF, et al. Pediatric invasive pneumococcal disease in Bolívar, Colombia. Infez Med. 2024;32(4):506–17.

Comment 5

Reviewer comment:

I understand that for safe handling of sensitive data, the hospital information cannot be accessed; however, in the methodology, they mention that case definitions were made, and then, based on this, inclusion and exclusion criteria were applied. I have two doubts about this: 1. The definition of case 2 includes unspecific symptoms of systemic infection that, in the age of the selected population, encompasses a broad spectrum of entities. It is also unclear whether the definition should include all symptoms or just one. Thus, is why it would be important to show how many cases were defined as case definition 1 o r case definition 2 and then which ones met the inclusion criteria. to modify the figure 1. In case you do not have or can not show the suggested data, explain better the screening and recruitment method. Were all patients who met a case definition (1 or 2) assessed to evaluate inclusion and exclusion criteria, and then it was defined that they entered the study?

Author response:

We revised the methodology to explain the two-step screening and diagnostic process. General physicians screened suspected cases using broad criteria (either otoscopic or systemic). All children who met these criteria were referred to an ENT specialist who confirmed AOM diagnosis using AAP standards. Only confirmed cases were assessed for eligibility. Unfortunately, the number of cases that met definition 1 or 2 separately was not recorded systematically. Figure 1 was not modified, but the recruitment explanation was clarified in the methods section.

Comment 6

Reviewer comment:
Regarding MEF sample collection, no references are cited to support the procedure used in this study. Is the sampling protocol proprietary? Was it adapted? I request the authors to provide further information in this regard.:

Author response:

We have now cited published methods to support the sample collection protocol (references 34 and 35).

34 Fekete S, Juhász J, Makra N, Dunai ZA, Kristóf K, Ostorházi E, et al. Characterization of middle ear microbiome in otitis media with effusion in Hungarian children: Alloiococcus otitidis may potentially hamper the microbial diversity. Heliyon [Internet]. 15 de noviembre de 2024;10(21):e39380. Disponible en: https://www.sciencedirect.com/science/article/pii/S2405844024154114

35. Marchisio P, Esposito S, Picca M, Baggi E, Terranova L, Orenti A, et al. Prospective evaluation of the aetiology of acute otitis media with spontaneous tympanic membrane perforation. Clin Microbiol Infect [Internet]. 1 de julio de 2017 [citado 5 de mayo de 2025];23(7):486.e1-486.e6. Disponible en: https://doi.org/10.1016/j.cmi.2017.01.010

Comment 7

Reviewer comment:
Since the authors' results suggest existing differences in the groups' characteristics, it would be worthwhile to analyze the difference through the appropriate statistical tests and add the p-value of this result to Table 1. This would evidence the differences.

Author response:

Thank you for your valuable observation. We agree that it would have been relevant to apply inferential statistics to compare the groups with respect to their sociodemographic characteristics. However, due to the small number of patients with *S. pneumoniae* (n = 6), the sample lacks sufficient statistical power to support meaningful comparisons. Therefore, we chose to present the data descriptively to avoid overinterpretation of underpowered analyses. Consequently, we also modified redaction in Results, in terms of not suggesting “differences” among groups: Line 241: “Among patients with AOM caused by microorganisms other than *S. pneumoniae*, thirty-three (60%) were male, with a median age of 12 months (IQR = 7 - 24).”

Comment 8

Reviewer comment:
There appears to be a difference in median age between patients with PNS and those with other causes, which should be discussed further. Could this be related to the increase in transient SPN colonization of the nasopharynx in school children?

Author response:

While children with *S. pneumoniae* were older, we cannot confirm statistical significance due to small sample size. We did run a Mann Whitney Test comparing age between the two groups and there was not statistical difference, however, we recognize the lack of power to ensure this result is not spurious. Therefore, we consider it not appropriate to make inferences about these differences in age.

Comment 9

Reviewer comment:

In the discussion, when referring to the vaccine recommendation, other types of vaccines, such as PCV10-SII (PNEUMOSIL) that protect against SPN serotypes 1, 5, 6A, 6B, 7F, 9V, 14, 19A, 19F, and 23F are not discussed. PCV-15 and PCV-20 are not mentioned either. Likewise, this recommendation does not make sense because the phenomenon of capsular replacement in other countries has already shown that once the vaccine is changed, the circulating serotypes begin to change in the following 5-10 years. Colombia started vaccination with PCV-13 3 years ago after many countries included PCV-15 in their programs. The discussion about the recommendation for the next vaccine should revolve around vaccines after PCV-13. Suggested references PMID (38336559, 39591182, 39153492)

Author response:

We have restructured discussion in regard to vaccine recommendations. We need, however, to recognize that PCV-13 is the current officially implemented vaccine in Colombia. Therefore, we framed our interpretation within this context.

In regard to other vaccines, we added a paragraph stating:

"As vaccine coverage expands to more serotypes, the replacement effect by non-vaccine serotypes (41) or other microorganisms will continue, as will the antimicrobial pressure due to the indiscriminate use of antibiotics. Among the cases of non-pneumococcal AOM, it was found that 25.5% of the participants received the PCV-13 vaccination schedule. This observation could serve as a baseline for future follow-ups to assess the

effect of replacement by other microorganisms and serotypes. Pneumococcal surveillance to non-invasive disease, including serotyping and resistance profiling, is critical for tracking the community-level circulation of multidrug-resistant strains. We acknowledge the development and licensure of newer pneumococcal conjugate vaccines (PCV10-SII, PCV-15 and PCV-20) which offer broader serotype coverage and are under evaluation or currently used in various immunization programs of other countries (49–51), but these vaccines were not yet licensed in Colombia at the time of this study."

Comment 10

Reviewer comment:

Although one of their important findings is that serotype 19a was found in a higher proportion, they do not discuss how, in Colombia, this circulating serotype is also the major cause of IPD PMID (34641809,32964223).

Author response:

Serotype 19A has emerged as a predominant serotype also in IPD. Its multidrug resistant pattern could be associated also to worse clinical outcomes such as IPD. We added a sentence to highlight this: "Evidence of a similar behavior in Colombia regarding isolates of S. pneumoniae causing IPD was found, wherein 19A and 6C are predominant serotypes."

Comment 11

Reviewer comment:
There are paragraphs in the discussion that merely mention facts but do not contradict or support the researchers' findings and do not contribute to the scope referred to in the objective. Examples of these are found in lines 344-347, 357-367, and 386-389.

Author response:

Thanks for your detailed revision of Discussion and meaningful suggestions. We revised the discussion and removed or integrated the identified sections into more cohesive and relevant paragraphs.

Comment 12

Reviewer comment:
I kindly suggest refining the information in the discussion to make the reader understand in a more adequate way the importance of the findings and how this study is the basis for further research on pneumococcal diseases different from IPD, as well as how the impact of the vaccination that has been applied to the Colombian context is reflected in its results and could be modified with the introduction of other vaccines.

Author response:

We reformulated the discussion to highlight key contributions: comparison with national studies, impact of vaccination on serotype distribution, the emergence of serotype 19A, and clinical aspects such as seasonality. Paragraphs were condensed to improve focus and readability.

Sincerely,

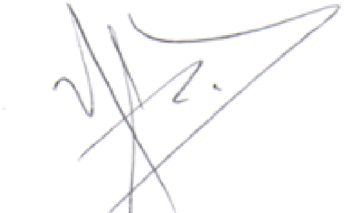

Nelson Alvis Guzman. PhD

Corresponding author
Universidad de la Costa
nalvis@cuc.edu.co
